# Smartphone-Based Approach-Avoidance Bias Modification Training for Depression: Randomized Clinical Trial

**DOI:** 10.2196/69033

**Published:** 2025-11-26

**Authors:** Maximilian Blomberg, Hilmar Gero Zech, Maximilian Kluge, Nico Böhmert, Helmut Platte, Timo Brockmeyer

**Affiliations:** 1 Department of Clinical Psychology and Translational Psychotherapy, Institute of Psychology, University of Münster, Fliednerstr. 21, Münster, 48149, Germany, 49 251-83-34190; 2 Department of Clinical Psychology and Psychotherapy, Institute of Psychology, University of Goettingen, Göttingen, Germany; 3 Department of Psychiatry and Psychotherapy, Technische Universität Dresden, Dresden, Germany; 4 Department of Child and Adolescent Psychiatry, Psychosomatics and Psychotherapy, Centre of Mental Health, University of Würzburg, Würzburg, Germany; 5 Paracelsus Roswithaklinik Bad Gandersheim, Bad Gandersheim, Germany

**Keywords:** affective disorders, approach bias, cognitive bias modification, eHealth, mHealth, smartphone intervention, mobile health

## Abstract

**Background:**

Effective treatments for depression are available, yet many patients do not respond to treatment or experience relapse. Cognitive bias modification aims to ameliorate cognitive biases that contribute to the development and maintenance of the disorder.

**Objective:**

This study examines the efficacy of a novel mobile approach-avoidance bias modification training with socioemotional cues for depression.

**Methods:**

In this randomized clinical superiority trial, 75 inpatients with depression underwent 6 sessions of either active or sham approach-avoidance bias modification training with socioemotional cues over the course of 2 weeks alongside inpatient treatment as usual. The primary outcome was self-reported depressive symptoms, and the secondary outcomes included approach-avoidance bias based on reaction time and response force, anhedonia, and positivity. Outcomes were assessed before and after the training, and again at 2-week and 6-month follow-ups. The primary hypothesis was that active training would lead to a stronger decrease in symptoms of depression at the end of training.

**Results:**

Both groups improved in depressive symptoms from baseline to the end-of-training assessment but did not differ in this regard (*B*=−1.14, 95% CI −5.65-3.41; *t*_188.61_=−0.47; *P*=.64; *d*=−0.09, 95% CI −0.46-0.28). Changes in anhedonia, positivity, and approach-avoidance bias were also not different between training groups, neither at end of training (*P*=.16) nor at the 2-week follow-up (*P*=.69). However, the active training group showed a significantly greater reduction in depressive symptoms from the baseline assessment to the 6-month follow-up (*B*=7.26, 95% CI 2.53-11.93; *t*_190.54_=2.95; *P*=.004; *d*=0.58, 95% CI 0.20-0.96). Permuted split-half reliability of the mobile assessment of approach-avoidance bias ranged from 0.77 to 0.94 for reaction times and from 0.81 to 0.93 for response force. Approach-avoidance bias was not altered by the training and did not mediate the training effects.

**Conclusions:**

Mobile approach-avoidance bias modification training with socioemotional cues did not reduce depressive symptoms in the short term but did in the long term. Mobile training and assessment versions may be more feasible in the future, as they require no joystick setup and can be conducted on patients’ smartphones. Future work needs to further examine short- and long-term efficacy and the mechanisms driving long-term symptom change in larger multicenter trials.

## Introduction

Major depressive disorder (MDD) is among the most prevalent mental disorders and a leading contributor to global disability [1] with patients facing a higher mortality rate [2], suffering from numerous somatic sequelae [3] and considerable psychosocial and socioeconomic challenges [4]. Besides medication, cognitive behavioral therapy (CBT) is considered the most well-established and empirically supported effective treatment for depression. However, as indicated by a comprehensive meta-analysis, CBT still yields only a 42% response rate and 36% remission following treatment [5].

The limited efficacy of treatment may in part be due to persisting cognitive biases that contribute to the development and maintenance of depression [6,7]. Cognitive biases are systematic biases in information processing [7], with memory [8], attention [9], and interpretation biases [10] most frequently studied. Furthermore, approach-avoidance biases describe automatic action tendencies following the evaluation of stimuli as either positive or negative [11,12]. Importantly, depression is characterized by reduced approach motivation toward positive cues [13]. For instance, contrary to healthy controls, patients show less approach behavior toward positive social-emotional cues (eg, smiling faces) and less avoidance of negative cues (eg, angry faces) [14].

Cognitive bias modification (CBM) aims to alter biased information processing by implementing contingencies into tasks traditionally used to assess these biases. For instance, approach-avoidance biases are often measured with a joystick task, where participants push or pull stimuli away or toward themselves [15]. To modify approach-avoidance tendencies, participants engage in a learning paradigm to consistently show approach or avoidance behavior in response to certain stimuli. In alcohol use disorder, patients repeatedly perform avoidance movements in response to alcohol-related cues and approach movements to nonalcoholic cues [16]. This active training is often compared to a sham version in which positive and negative stimuli are equally often approached and avoided [17].

Although approach-avoidance bias modification has shown promise in disorders such as addiction [18], eating disorders [19], and anxiety [20,21], evidence in depression remains limited. At least 2 randomized controlled trials have examined multisession training in inpatients with depressive symptoms. Vrijsen et al [22] reported that 4 biweekly sessions reduced depressive symptoms in patients with MDD compared to sham training, although the targeted bias was unchanged. Becker et al [23] found comparable effects in a mixed inpatient sample, but benefits were restricted to those with moderate-to-severe depression. In both studies, active training paired approach movements with positive stimuli and avoidance with neutral ones, while in the sham version, no systematic pairing of movements and stimuli was trained. Additionally, a small uncontrolled study in patients with depression found that combining 4 sessions of approach-avoidance training with behavioral activation was associated with improvements in depressive and anxiety symptoms as well as social connectedness [24]. Finally, a neuroimaging study using a single training session reported increased activation in reward-related regions (medial prefrontal cortex, caudate) during social reward anticipation, despite no change in bias [25].

All of these studies used a joystick task but varied in stimulus selection and training design. The first 2 studies utilized a broad spectrum of stimuli (eg, animals, people, objects) taken from the *International Affective Picture System* [26] with the aim to target general approach-avoidance biases [22,23], whereas the latter 2 focused on stimuli with positive and negative emotional facial expressions [24,25]. This emphasis on faces may be especially relevant, as depression is associated with altered approach-avoidance behavior toward emotional expressions [14] and less neural activation in reward circuitry during approach to social cues [27]. Training protocols also differed in the affective variety of stimuli included. In the first 2 studies, participants approached positive and avoided neutral stimuli [22,23], while the latter 2 combined an approach toward positive stimuli with lateral movements for neutral ones [24,25]. Evidence on working mechanisms of approach-avoidance training suggests that including avoidance of negative stimuli may further reduce dysfunctional biases [28]. Thus, paradigms integrating both approaches to positive and avoidance of negative stimuli may prove more effective than those focusing on only one of these.

Taken together, existing studies suggest that approach-avoidance training may serve as a useful add-on treatment for depression. However, evidence is currently limited by small samples [24,25]; missing control groups [24]; absent follow-ups [22,23,25]; mixed clinical samples [23]; single-session designs [25]; and issues of attrition, data loss, and variable intervals between baseline and end-of-training assessment [23].

This study seeks to evaluate a novel smartphone-based intervention derived from the *Mobile Approach-Avoidance Task* (mobile AAT) [29]. In this task, approach behavior is mapped as moving the smartphone (displaying the stimuli) toward the body. Correspondingly, avoidance behavior is mapped as moving the smartphone or the stimuli away from one’s own body. Compared to traditional joystick setups, the mobile format offers greater ecological validity, as it does not rely on zooming effects to simulate changes in physical distance to a stimulus [15] and as it allows training to be conducted flexibly in daily life. Furthermore, assessments of the mobile AAT have demonstrated adequate reliability of measurement using face stimuli [29,30]. In addition to reaction times, the mobile AAT also records response force in hand or arm movements. Reaction time and response forced approach-avoidance bias may capture distinct processes, as they were uncorrelated in earlier studies [29]. Given the absence of mediation effects for reaction time measures in earlier trials [22,23], exploring alternative indices such as response force may provide new insights into underlying mechanisms of approach-avoidance training.

Aside from changes in approach-avoidance tendencies, few studies have examined mediators that explain why depression symptoms may decrease after training. One candidate is anhedonia, a core feature of depression characterized by reduced motivation and diminished capacity to experience pleasure. Anhedonia predicts psychosocial functioning, depression severity, and treatment response [31,32] and has been proposed as a potential target of change in approach-avoidance bias modification [23]. Notably, it is associated with reduced approach motivation toward socioemotional cues [33]. We will therefore examine the effects of approach-avoidance training on anhedonia and its potential mediating role in symptom reduction. Conversely, positivity reflects a general tendency to view life, oneself, experiences, and the future positively. It is inversely related to depression [34,35] and aligns with behavioral activation approaches in social contexts [36]. Because depression is marked by reduced approach toward positive socioemotional cues [14], and the training targets this approach behavior, we will also examine its effects on positivity and its potential mediating role in symptom reduction.

The primary hypothesis in this study was that, in addition to CBT-based inpatient treatment as usual (TAU), 6 sessions of active approach-avoidance bias modification training would lead to a greater reduction in symptoms of depression than a sham training from baseline to end-of-training assessment. Additionally, we expected the active training group to be superior in symptom reduction from baseline to 2-week and 6-month follow-ups. Regarding secondary outcomes, we expected a stronger increase in approach-avoidance bias (ie, stronger approach of positive and stronger avoidance of negative stimuli) and positivity as well as a greater decrease in anhedonia in the active training group. Furthermore, we will test if a change in approach-avoidance bias from baseline to the end of training mediates the reduction in depressive symptoms. As exploratory aims, we examined the mediating roles of anhedonia and positivity and assessed whether training outcomes would be moderated by baseline characteristics.

## Methods

### Ethical Considerations

This monocenter randomized controlled superiority trial with 2 parallel arms was approved by the ethics committee of the Georg-Elias-Müller-Institute, University of Göttingen (#205) and registered at the German Clinical Trials Register (DRKS00022447). The study procedures adhered to the ethical standards of the institutional and national research committees and to the principles of the Declaration of Helsinki. All participants provided written informed consent and received financial compensation (€40 [US $45]) for their participation. All data were deidentified prior to analysis to ensure participant privacy and confidentiality.

### Design

Participants were randomly assigned (1:1 ratio) to receive either active or sham approach-avoidance training as an adjunct to inpatient TAU. Randomization was carried out with the *psych* package, version 2.2.9 [37]. In both conditions, participants were instructed that during the training, they would see facial expressions of varying emotions and would need to respond by either pulling the smartphone toward them or pushing it away. They were also told that the study would compare different training conditions and that they would receive more specific instructions on their respective condition after randomization. Depending on their allocated condition, one of two keywords (“depression1” or “depression2”) was entered by participants into the mobile AAT app, which was set up together with study personnel after screening for study eligibility. Participants were blinded to the keyword-condition mapping. However, the study personnel were not blinded to the keyword-condition mapping. All outcomes were either self-report measures or computerized tasks that were conducted independently and remotely by the patients without contact with the study personnel. Baseline, end-of-training, and 2-week follow-up data were collected through the mobile AAT app. Six-month follow-up assessments were conducted through the online survey framework *formr* [38]. Follow-up periods commenced after the end-of-training assessment. Patients were debriefed after the 6-month follow-up.

### Participants

Between October 2020 and December 2021, patients were recruited from the Paracelsus Roswithaklinik Bad Gandersheim, Germany, an inpatient rehabilitation center for mental and psychosomatic disorders with a focus on CBT. We collected data from a total of 75 participants. A total of 38 (51%) patients were randomly assigned to the active training and 37 (49%) to the sham training. Patients were initially made aware of the study with the help of leaflets, information events, and by their therapists at the end of group therapy sessions. Patients were eligible for the study if they were 18 years or older and met the diagnostic criteria for either (recurring) MDD, persistent depressive disorder, or both according to the *Diagnostic and Statistical Manual of Mental Disorders, Fifth Edition* (*DSM-5*) [39]. Study assessors screened patients using the *Structured Clinical Interview for DSM-5 Disorders* [40]. To maximize external validity, exclusion criteria were kept to a minimum of a remaining therapy duration of less than 14 days (to complete the training in addition to TAU), substance use disorder (nonabstinent), severe psychiatric comorbidity (eg, schizophrenia, bipolar disorder), medical instability, or acute suicidality. Data on ethnicity and socioeconomic status were not collected following principles of purpose limitation to the research questions and data minimization according to article 5 of the *General Data Protection Regulation* [41]. However, the vast majority of patients were White and stemmed from a Western European country.

Sample size was determined by an a priori power analysis using G*Power [42]. The study was powered for the primary hypothesis that the active training would lead to a stronger reduction in symptoms of depression from baseline to end-of-training assessment compared to the sham training. Based on an earlier study with a similar sample and a similar intervention [22], we expected a moderate within-between (time × group) interaction effect of *f*=.25 in a repeated-measures design, with *α*=.05 and statistical power 1-*β*=.95. While accounting for an approximate 20% of data loss (eg, due to technical errors), a minimum sample size of 65 would have been necessary, which was conservatively rounded up to 75, following recommendations for pilot trials [43].

### Treatments

#### Treatment as Usual

All participants received multimodal CBT-based TAU in an inpatient setting for a duration of 6 weeks. TAU comprised typical elements of inpatient psychotherapy such as group psychotherapy, individual psychotherapy, and involvement of caregivers as well as movement, occupational, and art therapy. Within these elements, CBT-oriented interventions were applied, such as psychoeducation, cognitive restructuring, behavioral activation, social skills and emotion regulation training, and relapse prevention. Testing the additive efficacy of CBM adjunct to TAU is a common design in this field of research [16,22,23,44].

#### Active Approach-Avoidance Training

Participants in the active training group received 6 sessions of an approach-avoidance training (20 min each) using an adapted version of the mobile AAT [29]. During the training, pictures of angry and happy faces taken from the *Radboud Faces Database* [45] appeared on the screen of the participants’ smartphones. A small cross or circle (approximately on the forehead) was superimposed on the face images. However, the superimposing was small enough so that the portrayed emotion could not be overlooked (see the study’s OSF repository [46] for an example). Participants were instructed to always pull the smartphone toward them if an image contained a cross and to always push their smartphone in response to images with circles. Each stimulus was preceded by a fixation cross displayed for 1.5 seconds [29]. Images were then presented for 2 seconds or until a response occurred.

In the active training group, all pictures of happy faces were paired with crosses and all angry faces with circles. Thus, participants learned to consistently approach happy faces and avoid angry ones (ie, to show valence-congruent movements). Each training session involved 40 images and 200 trials (10 female and 10 male faces, both in their respective happy and angry version, each repeated 5 times). Sessions were subdivided into 10 sets of 20 trials each. Before each training, participants completed 8 practice trials with different images to get accustomed to the movements and instructions.

#### Sham Approach-Avoidance Training

Participants in the sham training group received the same dosage and duration of training as participants in the active training group. The only difference was that in the sham training, the superimposing of crosses and circles on images was balanced, leading to 50% of trials demanding valence-congruent movements (approach happy or avoid angry faces) and the other half demanding valence-incongruent movements (approach angry or avoid happy faces).

#### Training Schedule

In both training conditions, participants were instructed to complete all sessions in the mobile AAT app (including baseline assessment, 6 training sessions, and end-of-training assessment) over the course of 2 weeks, with completing at most 1 session per day. We monitored adherence by regularly checking the completion of training sessions remotely and phoning participants in case of strong deviations from the training schedule (eg, multiple days without training session completion).

### Outcomes

#### Primary Outcome: Symptoms of Depression

Depressive symptoms were measured at baseline, the end of training, and 2-week and 6-month follow-ups by the *Beck Depression Inventory Second Edition* (BDI-II) [47], a very commonly used self-report questionnaire with good psychometric properties [48]. It comprises 21 items that assess the presence and severity of depressive symptoms over the last 2 weeks. Responses are given on a 4-point scale. A total score of 0‐13 represents minimal, 14‐19 mild, 20‐28 moderate, and 29‐63 severe depression. Values for Cronbach *α* at each assessment ranged from 0.90 to 0.94.

#### Secondary Outcomes

##### Approach-Avoidance Bias

Approach-avoidance bias was measured with the mobile AAT [29] at baseline, the end of training, and the 2-week follow-up. The source code of the mobile AAT is available on OSF [49]. For each assessment, trials were subdivided into 4 cells, corresponding to the combinations of the movement direction (approach vs avoid) and stimulus valence (happy vs angry faces). Following previous studies [22,23], scores for approach-avoidance bias were calculated as a compound measure by subtracting the median reaction times for valence-congruent movements from incongruent ones. According to Kahveci et al [50], this type of bias score should be called the double median difference score:


$$\begin{array}{l} Bias(RT)=\left(avoid\;positive+approach\;negative\right)\;\\ -\left(approach\;positive+avoid\;negative\right) \end{array}$$

Thus, a positive score indicates a relatively faster approach behavior toward happy faces and faster avoidance behavior in response to angry faces. Details on preprocessing the mobile AAT data can be found in Multimedia Appendix 1. In calculating the double median difference scores for response force, the equation above was changed to ensure that higher scores mirror a more pronounced reaction. This was achieved by subtracting the force of valence-incongruent movements from that of valence-congruent movements:


$$\begin{array}{l} Bias(Force)=\left(approach\;positive+avoid\;negative\right)\\ \;-\left(avoid\;positive+approach\;negative\right) \end{array}$$

Consequently, positive values indicate stronger responses (ie, more energetic arm movements) consistent with a “healthy” approach in response to happy faces and stronger avoidance movements in response to angry faces. In each assessment session, 60 pictures (15 female, 15 male faces, both in an angry and a happy version) from the *Radboud Faces Database* [45] were used. Assessment sessions consisted of 120 trials, divided into 2 blocks. In 1 block, participants were asked to perform valence-congruent movements (approach happy or avoid angry faces), while in the other block, they had to perform incongruent movements (approach angry or avoid happy faces). The order of blocks was randomized per participant and kept constant for the baseline and end-of-training assessments to prevent skewed bias measure changes due to habituation effects in the active training group. Thus, the assessment version of the AAT followed an explicit or feature-relevant design, instructing participants to approach or avoid faces depending on which emotion they express. Conversely, the training version of the AAT followed an implicit or feature-irrelevant design, instructing participants to show approach or avoidance movements depending on what symbol was superimposed on the face images.

##### Anhedonia

The *Dimensional Anhedonia Rating Scale* (DARS-17) [51] was used to assess anhedonia at baseline and end of training. The DARS-17 is a self-report measure consisting of 4 subscales measuring the enjoyment of pastimes and hobbies, food and drink, social activities, and sensory experiences. Each of the 17 items is rated on a 5-point Likert scale. Lower scores indicate stronger anhedonia. The reliability and validity of the German version have been demonstrated. Additionally, stronger anhedonia correlates with more severe symptoms of depression [51]. Values for Cronbach *α* at each assessment ranged from 0.86 to 0.94.

##### Positivity

Positivity was assessed by the *Positivity Scale* (P Scale) [34] at baseline and the end of training. The P Scale is an 8-item self-report questionnaire that measures the tendency to view oneself, life, and the future with a positive outlook. Items (eg, *I have great faith in the future*) are scored on a 5-point Likert scale, ranging from 1 (*strongly disagree*) to 5 (*strongly agree*). The total sum score correlates negatively with depression [34]. Values for Cronbach *α* at each assessment ranged from 0.85 to 0.90.

##### Credibility

To check how credible participants rated the training, we asked them at the 2-week follow-up to indicate on a 5-point Likert scale how efficacious they felt the intervention was, ranging from 1 (*very efficacious*) to 5 (*very inefficacious*), and how confident they were in their judgment, ranging from 1 (*very sure*) to 5 (*very unsure*).

### Statistical Analysis

Primary analysis followed an intention-to-treat approach. Outliers (|*z*|>3) were winsorized. Linear mixed-effects models were fitted with random intercepts for subjects and fixed effects of time, training condition, and their interaction [52]. Time was considered a repeated measures factor with either 4 (BDI-II), 3 (approach-avoidance bias), or 2 (DARS-17, P Scale) levels.

Interaction effects were tested using full versus reduced model comparisons [53], followed by post hoc comparisons per time interval (ie, baseline to end-of-training assessment, baseline to 2-wk follow-up, and baseline to 6-mo follow-up). Fixed effects were evaluated using restricted maximum likelihood and Satterthwaite approximation [54]. Effect sizes were calculated by dividing beta coefficients by the end-of-training SD in the sham training group [55]. Nonparametric bootstrapped 95% CIs were reported. Missing data were assessed using a formal test for missing completely at random and pattern-mixture models including missingness indicators [56].

To test potential moderators of training effects, we examined whether baseline outcomes influenced the interaction between time and training group. Specifically, we compared reduced models including time, training group, and their 2-way interactions with baseline outcomes with full models that additionally included the 3-way interaction between baseline outcome, training condition, and time. Additionally, we explored whether the effect of training condition on depressive symptoms at end-of-training, 2-week, and 6-month follow-ups was mediated by approach-avoidance bias, anhedonia, or positivity using path analysis. Baseline scores of the mediator and outcome were included as covariates [57]. Details on model specifications and used packages can be found in Multimedia Appendix 1.

Reaction times and response force were also analyzed at the trial level with maximal random effects structures [58]. Additionally, we conducted a complete case analysis and assessed permuted split-half reliability of the bias scores [59]. Lastly, we calculated proportions of clinically significant changes from the baseline assessment to the 6-month follow-up in the primary outcome between the groups (improvement in BDI-II scores ≥8 and final BDI-II at the 6-mo follow-up <19) [47,60].

## Results

### Sample Characteristics

Sample characteristics across conditions can be seen in Table 1. The training groups did not differ in any of these sample characteristics (*P*=.10).

**Table 1.** Sample characteristics at baseline (N=75).

Patient characteristic	Active training (n=38)	Sham training (n=37)	Total sample
Age (y), mean (SD)	49 (8.7)	48.8 (10.9)	48.9 (9.8)
Gender (women), n (%)	24 (63)	25 (68)	49 (65)
MDD^a^ (diagnosis), n (%)	32 (84)	32 (86)	64 (85)
Recurrent MDD	11 (29)	13 (35)	24 (32)
Double depression^b^	17 (45)	11 (30)	28 (37)
Persistent depressive disorder (diagnosis), n (%)	6 (16)	5 (14)	11 (15)
Psychotropic medication, n (%)	32 (84)	24 (65)	56 (75)
Inpatient treatment duration (d), mean (SD)	14.7 (5)	15.5 (8.1)	15.1 (6.7)

a MDD: major depressive disorder.

b Double depression: concurrent persistent depressive disorder and major depressive disorder.

Means, SD, and number of participants for primary and secondary outcomes at each assessment can be found in Table S1 in Multimedia Appendix 1. The training groups did not differ in any outcome at the baseline assessment (*P*=.22).

**Figure 1.**
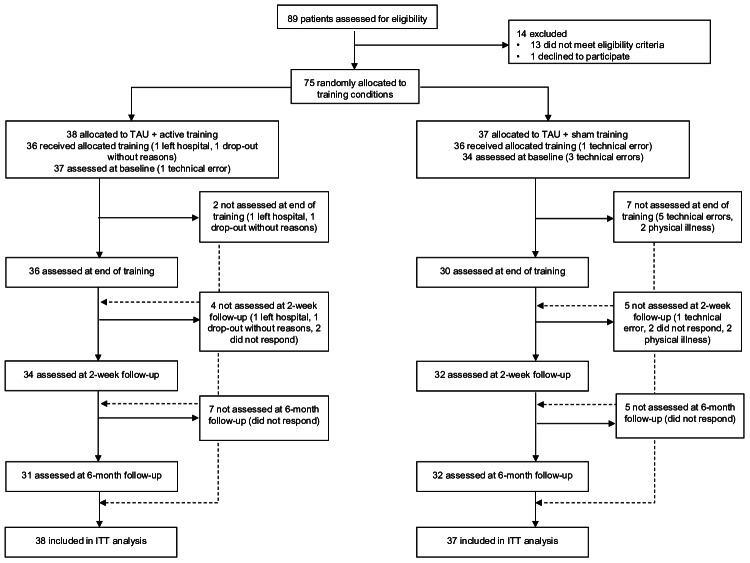
CONSORT (Consolidated Standards of Reporting Trials) flowchart. A patient may miss an earlier assessment while still completing a later one (see dashed lines). ITT: intention to treat; TAU: treatment as usual.

Figure 1 shows the participant flow. Participants in the active training condition received 5.8 (SD 0.6) training sessions on average. Likewise, participants in the sham training condition received 5.8 (SD 0.7) training sessions on average. Training dosage did not differ between groups (*t*_59.86_=0.15; *P*=.88). The training groups also did not differ in time spent on completing sessions (*t*_54.93_=−1.77; *P*=.08; active training: mean 10.3, SD 3.7 d; sham training: mean 12.2, SD 4.6 d). Reasons for training discontinuance were physical illness, early discharge, and technical errors using the smartphone app. At the 6-month follow-up, 7 (18%) patients were lost in the active training group and 5 (14%) in the sham training group. A total of 29 (76%) participants in the active training group completed all assessments, and 24 (65%) did so in the sham training group. Rates of complete and incomplete assessments did not differ between the groups (*χ*²_1.00_=0.70; *P*=.40). Among the 62 participants who provided the respective information at the 6-month follow-up, 2 participants in the active training group and 3 participants in the sham training group had started another inpatient treatment after discharge. A total of 24 participants in the active training group and 26 participants in the sham training group had started outpatient psychotherapy, and 15 patients in each training group changed psychotropic medication. Groups did not differ in any of these aspects (*P*=.75).

### Intention-to-Treat Analysis

**Figure 2.**
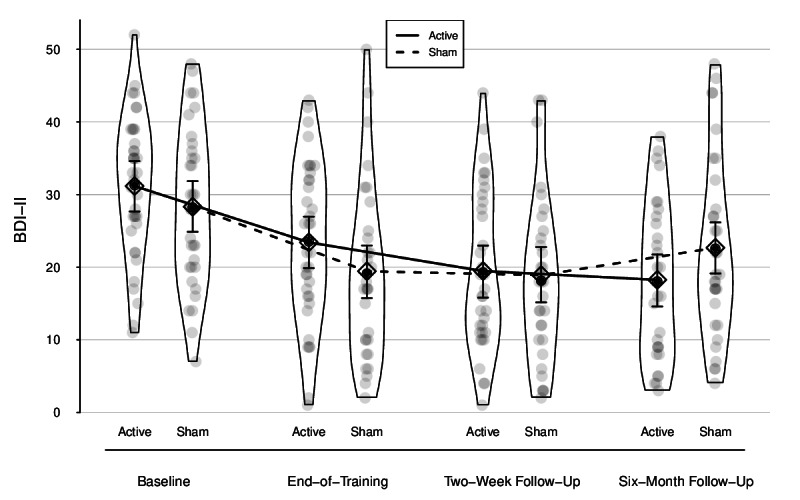
Model-based change trajectories in symptoms of depression. Gray dots represent individual data points. Solid black dots show empirical means. Diamonds show model-based estimates with 95% CIs. Bean width reflects distribution. BDI-II: Beck Depression Inventory II.

The full-reduced model comparison indicated that including training condition and its interaction with time significantly improved the model fit for symptoms of depression (*χ*²_4_=13.41; *P*=.009). Symptoms of depression decreased from baseline to the end-of-training assessment in the active training group, as indicated by a significant conditional main effect of time (*B*=−7.75, 95% CI −10.76 to −4.65; *t*_188.72_=−4.71; *P*<.001; *d*=−0.63, 95% CI −0.88 to −0.38). However, the interaction term for condition and time until the end-of-training assessment was not significant (*B*=−1.14, 95% CI −5.65 to 3.41; *t*_188.61_=−0.47; *P*=.64; *d*=−0.09, 95% CI −0.46 to 0.28). This means that the decrease in symptoms of depression was not different between the groups for the time period between baseline and the end of training. The same was true for the time between baseline and the 2-week follow-up assessment. Symptoms of depression decreased in the active training condition (*B*=−11.70, 95% CI −14.73 to −8.48; *t*_189.53_=−6.98; *P*<.001; *d*=−1.04, 95% CI −1.30 to −0.75), but this effect was not different from that in the sham training condition, as indicated by a nonsignificant interaction (*B*=2.27, 95% CI −2.40 to 6.88; *t*_190.15_=0.94; *P*=.35; *d*=0.20, 95% CI −0.21 to 0.61). At the 6-month follow-up, a conditional main effect indicated that symptoms of depression had decreased since the baseline assessment in the active training condition (*B*=−12.91, 95% CI −16.17 to −9.64; *t*_190.37_=−7.47; *P*<.001; *d*=−1.04, 95% CI −1.30 to −0.77). Furthermore, the interaction between condition and time of assessment was significant for this time period (*B*=7.26, 95% CI 2.53-11.93; *t*_190.54_=2.95; *P*=.004; *d*=0.58, 95% CI 0.20-0.96). This means that the decrease in symptoms of depression was significantly stronger for the active training group compared to the sham training group for the time between the baseline assessment and 6-month follow-up. Figure 2 displays the change trajectories for BDI-II scores by group, including both raw data and model-based estimates. Patient-level changes in symptoms of depression from baseline to 6-month follow-up are depicted in Figure S1 in Multimedia Appendix 1.

None of the full-reduced model comparisons for the secondary outcomes assessing approach-avoidance bias, general anhedonia, and “social activities” subscale as well as positivity were significant (*P*=.33). This indicates that changes over time were not different between training conditions for these outcomes. The pattern of the results remained consistent across other indices of approach-avoidance tendencies. This includes simple biases (differences in reaction time or response force during approach or avoidance of happy stimuli and differences in reaction time or response force during approach or avoidance of angry stimuli) and straightforward comparisons involving only reaction times and response forces for approaching happy stimuli and for avoiding angry stimuli. A significant conditional main effect indicated an increase in positivity from baseline to the end-of-training assessment in the active training group (*B*=1.94, 95% CI 0.65-3.21; *t*_64.11_=2.99; *P*=.004; *d*=0.32, 95% CI 0.11-0.53). However, this effect did not differ significantly from that observed in the sham training group (*B*=1.03, 95% CI −0.83 to 2.95; *t*_64.57_=1.07; *P*=.29; *d*=0.17, 95% CI −0.14 to 0.49). For feelings of anhedonia, neither the conditional main effect of time nor its interaction with the training group reached significance (all *P* values >.16). Similarly, for approach-avoidance bias, there was no significant conditional main effect of time in the active training group and no significant interaction with the training group, for either reaction times (*P*=.19) or response force (*P*=.23).

Table S2 in Multimedia Appendix 1 contains estimates and effect sizes for direct comparisons for all outcomes and assessment intervals, including conditional main effects of time in the active training condition and interaction effects. The number of measurements and participants in the random intercept models can be found in Table S3 in Multimedia Appendix 1. Figures S2-S5 in Multimedia Appendix 1 depict model-based change trajectories for secondary outcomes.

### Clinical Significance

Overall, 23 (31%) patients experienced a clinically significant change in BDI-II scores from baseline to the 6-month follow-up. In the active group, 15 (39%) patients showed a clinically significant improvement. In the sham training group, 8 (41%) patients did so, which corresponds to a number needed to treat [61] of 5.6 or an area under the receiver operating characteristic curve of 0.59% [62]. However, the training groups did not differ significantly in clinical significant change (*χ*²_1.00_=2.03; *P*=.15).

### Reliability of Bias Assessment

The Spearman-Brown permuted split-half reliabilities for measuring approach-avoidance bias at different assessments with the mobile AAT ranged from 0.77 to 0.94 for reaction times and from 0.81 to 0.93 for response force. This indicates acceptable-to-excellent reliability across the 2 forms of measuring approach-avoidance bias across time [63].

### Credibility

At the 2-week follow-up, the training groups did not differ in how they perceived the effectiveness of the intervention (*t*_63.65_=1.15; *P*=.26; active training: mean 3.4, SD 1.0; sham training: mean 3.1, SD 0.9) nor in the confidence of their judgment of the efficacy of the intervention (*t*_63.67_=–0.22; *P*=.83; active training: mean 3.3, SD 1.0; sham training: mean 3.4, SD 0.9).

### Moderation Analysis

Full-reduced model comparisons showed that baseline BDI-II levels significantly moderated the interaction between training condition and time on the approach-avoidance bias based on response force. Specifically, the bias increased more strongly in the active training condition among participants with higher baseline depression. However, this effect did not remain significant after Bonferroni correction. No other moderation effects were observed. The results of the moderation analysis can be found in Table S7 in Multimedia Appendix 1.

### Mediation Analysis

The analysis of the effect of training condition on symptoms of depression at the 6-month follow-up via approach-avoidance bias based on reaction time at the end of training including the baseline levels of symptoms of depression and the bias as covariates in a path analysis with 10,000 bootstrapped resamples revealed no significant mediation effect (indirect effect −0.06, 95% CI −1.71 to 1.22; *P*=.91). The same was true for any other potential mediator including anhedonia, positivity, and approach-avoidance bias as response force at the end-of-training assessment and follow-ups (*P*=.17).

### Further Analysis

The results of maximum random effects models, missing data analysis, and complete case analyses all resembled the main findings and can be found in Multimedia Appendix 1.

## Discussion

To the best of our knowledge, this is the first clinical trial to examine mobile approach-avoidance training adjunct to inpatient TAU in depression. In contrast to our primary hypothesis, the active training was not superior to the sham version in short-term reductions in depressive symptoms (ie, at the end of training and 2-wk follow-up). However, active approach-avoidance training was superior to sham training regarding long-term reductions in depressive symptoms (ie, at the 6-mo follow-up). In terms of clinically significant change, when comparing a patient who received active training against a patient who received sham training, 59% of the time, the patient who received the active training would have a better response (ie, a clinically significant change). This resembles a small-to-moderate effect size [62], although this difference was not significant, potentially due to the sample size.

The lack of short-term superiority of the intervention is in contrast to a previous study on approach-avoidance training in inpatients with depression [22] and a similar trial in a transdiagnostic inpatient sample with elevated symptoms of depression [23]. These studies used joystick-based training instead of a mobile version. However, it does not seem likely that this would lead to faster clinical improvements. A simple reason could be that the short-term effects became visible in the larger samples of these 2 previous studies. In contrast, the result of the long-term efficacy of the training in the present study extends findings from an uncontrolled proof-of-concept study [24], in which clinical improvements were observed 3 months after approach-avoidance training combined with behavioral activation. Furthermore, the long-term efficacy of the training in depression aligns with the positive outcomes of the intervention observed in alcohol-use disorder, where studies have repeatedly demonstrated significant improvements at 1-year follow-ups for individuals undergoing approach-avoidance training [16,18,44]. Given that previous studies on approach-avoidance training in inpatients with depression did not assess long-term effects [22,23], this study offers valuable new insights into the efficacy of this intervention over an extended time period. Furthermore, this study is the first to demonstrate the feasibility of a mobile-based approach-avoidance training that can be easily integrated into patients’ daily lives, requiring only a smartphone and about 15 minutes per session.

This study also examined potential mechanisms of approach-avoidance training. However, the findings on secondary outcomes showed no differential effects of active training on anhedonia, positivity, or reaction-time and response-force biases, where both training groups improved similarly. This aligns with previous studies of approach-avoidance training in depression and other mental disorders, including addiction [16,22,23]. Likewise, the reduction in depressive symptoms was not mediated by changes in bias. Thus, the underlying mechanisms of the intervention remain to be elucidated in future research. In this respect, approach-avoidance training aligns with other interventions, such as antidepressants and CBT, for which the exact mechanisms are still not fully understood [64].

However, several methodological aspects must be considered when interpreting our results. In the bias assessment, participants were instructed to approach or avoid faces based on their emotional expression (feature-relevant). In contrast, during training, responses were directed by superimposed symbols, rendering the task feature-irrelevant to the depicted emotion, although the emotional content remained visible. This implicit design was chosen to align with prior clinical intervention studies [16,22,23]. However, approach-avoidance behavior may differ between explicit, feature-relevant tasks and implicit, feature-irrelevant tasks [65]. Developing a feature-relevant training version in the future could therefore be informative, although creating a comparable control condition would be challenging, as it would require varying task instructions throughout the training. Finally, we retained a feature-relevant assessment version to maximize reliability and comparability with earlier mobile AAT studies [29,30]. Indeed, evidence indicates that feature-relevant instructions yield more reliable and valid measures than feature-irrelevant paradigms [50,66].

What are the possible mechanisms of the training that could lead to a long-term reduction in symptoms of depression? Changes in implicit approach tendencies, particularly toward socioemotional cues, may gradually translate into explicit social approach behaviors. Over time, such changes can reduce depressive symptoms by fostering rewarding social experiences. Dysfunctional processing of socioemotional stimuli often leads to social withdrawal, limiting positive reinforcement and thereby maintaining depression [13,67,68]. Consistent with this, the quality of social relationships and behavioral activation are key determinants of symptom severity and chronicity [69,70]. An encouraging social approach is therefore a central component of contemporary psychotherapy [71]. Our study extends previous approach-avoidance training in depression [22,23], which typically used heterogeneous image sets [26], by employing standardized facial expressions. This design specifically targets impaired approach behavior in social contexts [14,65]. Furthermore, functional magnetic resonance imaging findings suggest that approach-avoidance training can normalize aberrant reward sensitivity to positive social cues [25,27]. Training approach behavior toward positive stimuli could also strengthen mood-incongruent processing, which is diminished in depression [72,73], yet has been shown to repair negative mood states [74] and predict more favorable outcomes [75]. Individuals may need time and opportunities after inpatient treatment to translate implicit training gains in social approach behavior into real-world social interactions for these effects to unfold gradually. Future research should therefore examine downstream effects of approach-avoidance training on social connectedness, relationship functioning, and behavioral activation as potential mediators of long-term symptom reduction [36].

Patients with depression frequently relapse or experience symptom worsening after treatment [5]. Consistent with this, depressive symptoms in the sham training group increased from the 2-week to 6-month follow-up. Such long-term limitations of treatment may partly reflect persistent cognitive biases that contribute to the maintenance of depression [6,7] and were directly targeted in the active training condition. Thus, our findings may also suggest that active approach-avoidance training helped prevent symptom worsening from mid- to long-term. Future studies should include longer follow-ups (eg, up to 12 mo, as is common in addiction research [16]) to clarify the trajectory of symptom change over time.

Our study’s findings should be considered in light of its strengths and limitations. A strength of the mobile AAT is that it enables assessment of both reaction times and response force, adding a dimension not available in traditional joystick paradigms. However, mobile AAT paradigms are still relatively new [29], and prior studies have struggled to reliably detect force-related approach-avoidance biases [76,77]. More basic research is needed to clarify the nature of force-based biases and their relation to reaction times. Additionally, we were able to assess the vast majority (n=63, 84%) of patients at the 6-month follow-up. Dropout and attrition rates were considerably low and did not differ between conditions, but still, some data were lost due to technical errors. A large number (n=50) of the participants had started outpatient psychotherapy by the 6-month follow-up. Importantly, this rate did not differ between the training groups. This aligns with the general logic of randomized clinical trials, in which randomization is assumed to balance potential confounding factors (eg, concurrent outpatient therapy) across training conditions [78]. Due to the monocentric approach in a clinic in a Western European country, the generalizability of the findings to other treatment settings and differing cultural contexts is limited. Patients completed the mobile AAT independently on their smartphones, and we could not fully verify whether prototypical approach-avoidance movements were performed throughout. We monitored adherence by tracking session completion online and phoning participants if deviations occurred. Although patients could technically complete multiple training sessions per day, data indicate that they generally followed the 2-week protocol, and training duration did not differ between the groups. Still, future training versions may technically be restricted to 1 session per day. The results of the credibility checks do not suggest that patients in the 2 training conditions differed in their expectations regarding the efficacy of the training 2 weeks after completion of the intervention. The clinical outcomes of our study relied on self-reported measures. Future research may also include clinician-rated assessments of depressive symptoms to support the self-assessment. Furthermore, our study utilized a limited range of highly standardized stimuli with facial expressions of emotions from a well-established image database [45]. While using stimuli of friendly and angry faces might appear overly simplistic, customizing the training with individualized stimuli for each patient could hinder the standardization of the intervention and introduce confounding variables. This needs to be considered when assessing the reliability of the approach-avoidance task. Finally, our sample size was sufficient to detect moderate interaction effects, but it lacked power to identify smaller treatment effects. A larger sample would increase sensitivity to subtle short-term effects, yield more precise estimates of change over time, and allow adequately powered moderation and mediation analyses, which in this study were only exploratory.

This study investigated the efficacy of a mobile approach-avoidance training as an add-on to inpatient treatment for individuals with depression. Despite its limitations, the study used novel methodological approaches that could pave the way for more flexible research designs for CBM studies in the future. In summary, we found evidence for long-term but not short-term superiority of the active training compared to a sham version in terms of the primary outcome, symptoms of depression. This effect, however, cannot yet be explained by a change in approach-avoidance bias, neither when measured via reaction times nor when measured via response force. Future research should aim to elucidate the mechanisms at work of this intervention and replicate the findings in larger samples in multicenter trials.

## Supplementary material

10.2196/69033 Multimedia Appendix 1 Supplementary material including additional tables, figures, and details of statistical analyses.

10.2196/69033 Checklist 1 CONSORT-EHEALTH checklist (V1.6.1).

## Abbreviations

BDI-II: Beck Depression Inventory Second Edition
CBM: cognitive bias modification
CBT: cognitive behavioral therapy
DARS-17: Dimensional Anhedonia Rating Scale
*DSM-5*: *Diagnostic and Statistical Manual of Mental Disorders, Fifth Edition*
MDD: major depressive disorder
mobile AAT: Mobile Approach-Avoidance Task
OSF: Open Science Framework
P Scale: Positivity Scale
TAU: treatment as usual
