# Supplementary material for: Smartphone-Based Approach-Avoidance Bias Modification Training for Depression: Randomized Clinical Trial
Source: JMIR Mhealth Uhealth. 2025 Nov 26;13:e69033. doi: 10.2196/69033 (PMC12661601; doi:10.2196/69033)
Supplement: Multimedia Appendix 1 [file mhealth-v13-e69033-s001.pdf]

Supplement to the Study "Smartphone-Based Approach-Avoidance Bias Modification Training for Depression - A Randomized Clinical Trial"

Methods

Details on Preprocessing of Mobile AAT Data

In accordance with the preprocessing procedures outlined for the mobile AAT (Zech et al., 2022), median reaction times were calculated based solely on correct response trials, provided that the participant demonstrated a correct response in at least 70% of the trials (e.g., an approach response in an approach trial). Consistent with previous studies and preprocessing recommendations for approach-avoidance data (Kahveci et al., 2023; Zech et al., 2020, 2022), trials with reaction times less than 200 ms or more than 2000 ms, as well as those exceeding two standard deviations above or below the mean reaction time/response force, were discarded. The same criteria for the validity of trials and their aggregation were used for the preprocssing of the response force data. Furthermore, we computed simple bias scores for each valence category individually by determining the differences in reaction time associated with approach and avoidance movements.

Details on Statistical Analysis

Linear mixed-effects models were implemented in *R* (Team, 2020) using the *lme4* package (Bates et al., 2014), following recommendations by (Baayen et al., 2008) for modeling repeated measures with random intercepts. Fixed effects were evaluated using the *lmerTest* (Kuznetsova et al., 2017) package with REML estimation and Satterthwaite approximation (Luke, 2017). Effect sizes were calculated based on recommendation by

(Feingold, 2009), dividing model coefficients by the standard deviation at end-of-training in the control group. Confidence intervals (95%) were computed using 1000 bootstraps (Davison & Hinkley, 1997). Missing data were examined with Little's MCAR test (Little, 1988) and addressed through pattern-mixture models that included missingness as a fixed effect (Hedeker & Gibbons, 1997), using likelihood ratio tests for comparison. Missingness was defined per participant as at least one missing observation in the primary outcome (BDI-II) across assessments. We also conducted a complete case analysis that included participants for whom data were available at all assessments. Mediation analysis was performed using the mediation package (Tingley et al., 2014), controlling for baseline values (Hayes & Rockwood, 2017). Trial-level analyses of reaction times and force used models with maximal random effects structures, as recommended by (Barr et al., 2013). This analysis method can accurately account for the complex data structure in assessing approach-avoidance tendencies, which involves multiple layers of nesting. For example, a single participant might see various faces from the same actor, each displaying different emotions and paired with different movements, and these encounters occur at different assessments. We assessed the reliability of measurement of approach-avoidance bias based on double median difference scores using permuted Spearman-Brown split-half reliability (Kahveci et al., 2025; Zech et al., 2020) with 2000 permutations. Additionally, we explored moderation of interaction effects between condition and time by baseline outcomes (see below).

Moderation Models

To test potential moderators of training effects, we examined whether baseline outcomes influenced the interaction between time and training group. Specifically, we compared reduced models including time of assessment, training group, and their two-way interactions with baseline outcomes with full models that additionally included the three-way interaction between baseline outcome, training condition, and time:

$$\text{Reduced model:} \quad Outcome_i \sim Condition * Time + Outcome_{j,baseline} + 1 \mid ID + Outcome_{j,baseline} : Condition + Outcome_{j,baseline} : Time$$

$$\text{Full model:} \quad Outcome_i \sim Condition * Time * Outcome_{j,baseline} + 1 \mid ID$$

Each outcome analysis was crossed with all baseline measures. For example, we tested whether baseline BDI-II, Bias-RT, Bias-Force, anhedonia, moderated the interaction of condition and time on BDI-II outcomes.

Mediation Models

Additionally, we explored whether the effect of training condition on depressive symptoms at end-of-training, two-week, and six-month follow-ups was mediated by approach–avoidance bias, anhedonia, or positivity using path analysis. Baseline scores of the mediator and outcome were included as covariates (Hayes & Rockwood, 2017):

$$\text{Outcome model:} \quad Outcome_i \sim Condition + Mediator_j + Outcome_{i,baseline} + Moderator_{j,baseline}$$

$$\text{Mediator model:} \quad Mediator_j \sim Condition + Moderator_{j,baseline}$$

Importantly, outcomes could only be mediated by variables assessed at the same time or earlier. For example, the effect of training condition on end-of-training BDI-II scores could be mediated by approach–avoidance bias measured at end-of-training, but not by bias scores from the two-week follow-up.

Software & Package Versions

- lme4: 1.1.37
- lmerTest: 3.1.3
- mediation: 4.5.1
- R version 4.5.1 (2025-06-13 ucrt)
- G*Power: 3.1 (Faul et al., 2009)

Results

Maximum-Random-Effects Models

Results from the maximum-random-effects models for reaction times and response force did not reveal a change in the approach-avoidance bias either. For reaction times, the highest order significant effect was a conditional main effect for stimulus emotion, indicating faster reaction times for happy vs. angry faces irrespective of approach vs. avoidance movements, $t(31.92) = 2.11$, $p = .043$. Furthermore, a conditional main effect for movement indicated faster reaction times for approach vs. avoidance movements irrespective of the emotion of the stimulus, $t(34.88) = 2.74$, $p = .010$. Regarding response force, the highest order significant effect was the interaction between movement and condition that indicated a general difference in applied force for avoidance movements between groups, irrespective of time of assessment or stimulus emotion, $t(40.00) = 2.26$, $p = .029$.

Missing Data Analysis

For all outcomes, likelihood ratio tests indicated that the pattern of results did not differ when including missingness into the models (all ps $> .230$, see Supplement, Table S4). The results of the pattern mixture models were supported by Little's MCAR test, $\chi^2(177) = 194.50$, $p = .175$, indicating that data were missing completely at random.

Complete Case Analysis

Without exception, the pattern of results of the complete case analysis matched those of the ITT analysis (see Tables S5 and S6 and Figures S7-S11 in the Supplement).

Robustness Check: Selective Dropout

To address the possibility that the six-month effect was driven by selective dropout, we reran the analysis excluding participants with missing BDI-II scores at the six-month follow-up. As shown in the table below, results were unchanged, with the only significant interaction effect observed for BDI-II at six months:

Estimates and Effect Sizes for Direct Comparisons between Training Conditions with 95% Confidence Interval in the Dropout Analysis

Outcome	Time (End-of-Training)		Sham x Time (End-of-Training)		Time (Two-Week Follow-Up)		Sham x Time (Two-Week Follow-Up)		Time (Six-Month Follow-Up)		Sham x Time (Six-Month Follow-Up)	
	B	d	B	d	B	d	B	d	B	d	B	d
BDI-II	-6.80 [-10.38, -3.22]	-0.56 [-0.86, -0.27]	-2.23 [-7.27, 2.93]	-0.18 [-0.60, 0.24]	-11.02 [-14.58, -7.67]	-0.91 [-1.20, -0.63]	1.74 [-3.37, 6.62]	0.14 [-0.28, 0.55]	-12.31 [-15.99, -8.60]	-1.01 [-1.32, -0.71]	6.69 [1.92, 11.79]	0.55 [0.16, 0.97]
Bias-RT	29.25 [-15.15, 79.17]	0.29 [-0.15, 0.79]	-50.72 [-117.73, 16.75]	-0.51 [-1.17, 0.17]	30.17 [-15.63, 74.75]	0.30 [-0.16, 0.75]	-4.42 [-71.84, 65.09]	-0.04 [-0.72, 0.65]				
Bias-Force	-0.89 [-3.15, 1.24]	-0.26 [-0.91, 0.36]	2.06 [-0.92, 5.39]	0.60 [-0.27, 1.56]	0.58 [-1.68, 2.75]	0.17 [-0.49, 0.80]	0.57 [-2.69, 3.81]	0.17 [-0.78, 1.10]				
DARS-17	2.03 [-2.33, 6.45]	0.14 [-0.16, 0.45]	4.36 [-2.10, 10.14]	0.30 [-0.15, 0.71]								
P Scale	1.64 [0.18, 3.05]	0.28 [0.03, 0.53]	1.60 [-0.43, 3.51]	0.27 [-0.07, 0.60]								

Note. BDI-II = Beck Depression Inventory II; Bias-RT = Approach-avoidance Bias (Reaction Time); Bias-Force = Approach-avoidance Bias (Response Force); DARS-17 = Dimensional Anhedonia Rating Scale 17; P Scale = Positivity Scale

Difference of Bias against Zero

At baseline, the reaction-time–based approach–avoidance bias did not differ from zero, $t(58.00) = 1.80$, $p =$ .077. In contrast, the bias was significantly different from zero at end-of-treatment and two-week follow-up, $t(55.00) = 2.06$, $p = .044$ and $t(49.00) = 3.70$, $p < .001$, respectively. This indicates that participants showed no bias at baseline, i.e. no preference for approaching positive/avoiding negative stimuli over approaching negative/avoiding positive stimuli. Over time, however, the bias shifted in the expected direction (greater approach of positive and/or avoidance of negative stimuli). Importantly, and already shown by the main analysis, change in approach-avoidance bias did not differ between training conditions.

For the response-force–based approach–avoidance bias, results indicated stronger approach-negative/avoid-positive behavior at baseline $t(58.00) =$ -2.08, $p = .042$ and at end-of-training $t(55.00) =$ -2.11, $p = .039$. At the two-week follow-up, however, the bias was no longer significantly different from zero, $t(49.00) = 0.21$, $p = .831$. As shown in the main analysis, change in approach-avoidance bias did not differ between training groups.

Additional Tables & Figures

Table S1

Means and Standard Deviations across Training Groups and Assessments

Outcome		Baseline		End-of-Training		Two-Week Follow-Up		Six-Month Follow-Up	
		Active	Sham	Active	Sham	Active	Sham	Active	Sham
	n	37	34	36	30	34	32	31	32
BDI-II	M	31.30	28.24	23.53	19.13	19.29	18.16	18.03	22.56
	SD	9.73	10.85	10.98	12.25	10.95	11.30	10.60	12.46
	n	31	28	31	25	29	21	0	0
Bias-RT	M	11.74	50.81	32.79	24.82	34.17	60.81		
	SD	123.38	134.47	112.78	99.52	90.17	81.43		
	n	31	28	31	25	29	21	0	0
Bias-Force	M	-0.83	-2.41	-0.73	-0.89	0.48	-0.44		
	SD	3.30	7.70	2.37	3.38	2.83	3.58		
	n	37	34	36	29	0	0	0	0
DARS-17	M	39.84	36.68	42.28	44.69				
	SD	13.06	12.43	15.75	13.81				
	n	37	34	36	29	0	0	0	0
P Scale	M	21.11	21.38	23.06	24.28				
	SD	6.11	5.13	6.00	6.00				

Note. BDI-II = Beck Depression Inventory II; Bias-RT = Approach-avoidance Bias (Reaction Time); Bias-Force = Approach-avoidance Bias (Response Force); DARS-17 = Dimensional Anhedonia Rating Scale 17; P Scale = Positivity Scale

Table S2

Estimates and Effect Sizes for Direct Comparisons between Training Conditions with 95% Confidence Interval in the ITT Analysis

Outcome	Time (End-of-Training)		Sham x Time (End-of-Training)		Time (Two-Week Follow-Up)		Sham x Time (Two-Week Follow-Up)		Time (Six-Month Follow-Up)		Sham x Time (Six-Month Follow-Up)	
	B	*d*	B	*d*	B	*d*	B	*d*	B	*d*	B	*d*
BDI-II	-7.75 [-10.76, -4.65]	-0.63 [-0.88, -0.38]	-1.14 [-5.65, 3.41]	-0.09 [-0.46, 0.28]	-11.70 [-14.73, -8.48]	-0.95 [-1.20, -0.69]	2.27 [-2.40, 6.88]	0.19 [-0.20, 0.56]	-12.91 [-16.17, -9.64]	-1.05 [-1.32, -0.79]	7.26 [2.53, 11.93]	0.59 [0.21, 0.97]
Bias-RT	22.56 [-16.58, 63.60]	0.23 [-0.17, 0.64]	-41.05 [-99.30, 15.04]	-0.41 [-1.00, 0.15]	26.13 [-17.08, 68.65]	0.26 [-0.17, 0.69]	-3.35 [-66.83, 57.16]	-0.03 [-0.67, 0.57]				
Bias-Force	0.11 [-1.88, 2.15]	0.03 [-0.56, 0.64]	1.41 [-1.51, 4.24]	0.42 [-0.45, 1.26]	1.30 [-0.63, 3.25]	0.39 [-0.19, 0.96]	0.65 [-2.38, 3.71]	0.19 [-0.70, 1.10]				
DARS-17	2.89 [-1.24, 7.21]	0.21 [-0.09, 0.52]	4.57 [-1.60, 10.21]	0.33 [-0.12, 0.74]								
P Scale	1.94 [0.65, 3.21]	0.32 [0.11, 0.53]	1.03 [-0.83, 2.95]	0.17 [-0.14, 0.49]								

Note. BDI-II = Beck Depression Inventory II; Bias-RT = Approach-avoidance Bias (Reaction Time); Bias-Force = Approach-avoidance Bias (Response Force); DARS-17 = Dimensional Anhedonia Rating Scale 17; P Scale = Positivity Scale

Table S3

Number of Measurements and Patients in the Random Intercept Models

Outcome	Number of measurements	Number of patients
BDI-II	266	75
Bias-RT	165	70
Bias-Force	165	70
DARS-17	136	72
P Scale	136	72

Note. BDI-II = Beck Depression Inventory II; Bias-RT = Approach-avoidance Bias (Reaction Time); Bias-Force = Approach-avoidance Bias (Response Force); DARS-17 = Dimensional Anhedonia Rating Scale 17; P Scale = Positivity Scale

Table S4

Effects of Complete Case Status on Outcomes: Comparison of the Standard Model and a Pattern Mixture Model

Outcome	df	Chisq	p
BDI-II	1	1.44	.230
Bias-RT	1	1.02	.312
Bias-Force	1	0.14	.708
DARS-17	1	0.06	.803
P Scale	1	0.69	.406

Note. LR = likelihood ratio; BDI-II = Beck Depression Inventory II; Bias-RT = Approach-avoidance Bias (Reaction Time); Bias-Force = Approach-avoidance Bias (Response Force); DARS-17 = Dimensional Anhedonia Rating Scale 17; P Scale = Positivity Scale

Table S5

Estimates and Effect Sizes for Direct Comparisons between Training Conditions with 95% Confidence Interval in the Complete Case Analysis

Outcome	Time (End-of-Training)		Sham x Time (End-of-Training)		Time (Two-Week Follow-Up)		Sham x Time (Two-Week Follow-Up)		Time (Six-Month Follow-Up)		Sham x Time (Six-Month Follow-Up)	
	B	*d*	B	*d*	B	*d*	B	*d*	B	*d*	B	*d*
BDI-II	-7.07 [-10.66, -3.61]	-0.69 [-1.05, -0.35]	-1.14 [-6.00, 4.40]	-0.11 [-0.59, 0.43]	-11.28 [-15.08, -7.73]	-1.11 [-1.48, -0.76]	3.15 [-2.22, 8.59]	0.31 [-0.22, 0.84]	-12.07 [-15.83, -8.50]	-1.18 [-1.55, -0.83]	8.61 [3.58, 14.03]	0.84 [0.35, 1.38]
Bias-RT	32.05 [-12.43, 73.69]	0.34 [-0.13, 0.79]	-43.26 [-105.48, 23.83]	-0.46 [-1.13, 0.25]	31.61 [-14.37, 75.42]	0.34 [-0.15, 0.81]	-0.22 [-70.26, 69.12]	-0.00 [-0.75, 0.74]				
Bias-Force	-1.02 [-3.04, 1.21]	-0.29 [-0.85, 0.34]	3.13 [-0.06, 6.12]	0.88 [-0.02, 1.72]	0.48 [-1.41, 2.56]	0.13 [-0.40, 0.72]	1.61 [-1.57, 4.89]	0.45 [-0.44, 1.38]				
DARS-17	2.66 [-1.95, 7.14]	0.21 [-0.15, 0.56]	2.64 [-4.22, 9.61]	0.21 [-0.33, 0.75]								
P Scale	1.55 [0.22, 2.95]	0.28 [0.04, 0.53]	1.91 [-0.30, 4.13]	0.34 [-0.05, 0.74]								

Note. BDI-II = Beck Depression Inventory II; Bias-RT = Approach-avoidance Bias (Reaction Time); Bias-Force = Approach-avoidance Bias (Response Force); DARS-17 = Dimensional Anhedonia Rating Scale 17; P Scale = Positivity Scale

Table S6

Number of Measurements and Patients in the Random Intercept Models in the Complete Case Analysis

Outcome	Number of measurements	Number of patients
BDI-II	212	53
Bias-RT	135	52
Bias-Force	135	52
DARS-17	105	53
P Scale	105	53

Note. BDI-II = Beck Depression Inventory II; Bias-RT = Approach-avoidance Bias (Reaction Time); Bias-Force = Approach-avoidance Bias (Response Force); DARS-17 = Dimensional Anhedonia Rating Scale 17; P Scale = Positivity Scale

Table S7

Moderating Influences of Baseline Values on the Training Effect on Outcomes: Full-reduced Model Comparisons

Outcome (baseline)	BDI-II			Bias-RT			Bias-Force			DARS-17			P Scale		
	Chisq	*df*	*p*	Chisq	*df*	*p*	Chisq	*df*	*p*	Chisq	*df*	*p*	Chisq	*df*	*p*
BDI-II	3.00	2.19	.533	2.00	3.62	.164	2.00	8.92	.012*	1.00	1.36	.243	1.00	0.01	.910
Bias-RT	3.00	3.85	.279	2.00	0.75	.688	2.00	1.79	.409	1.00	1.49	.223	1.00	2.27	.132
Bias-Force	3.00	5.69	.128	2.00	3.53	.171	2.00	2.88	.237	1.00	3.16	.075	1.00	0.07	.794
DARS-17	3.00	5.99	.112	2.00	0.82	.665	2.00	2.30	.316	1.00	0.04	.838	1.00	0.11	.740
P Scale	3.00	6.37	.095	2.00	0.03	.985	2.00	3.58	.167	1.00	2.18	.140	1.00	1.81	.178

Note. BDI-II = Beck Depression Inventory II; Bias-RT = Approach-avoidance Bias (Reaction Time); Bias-Force = Approach-avoidance Bias (Response Force); DARS-17 = Dimensional Anhedonia Rating Scale 17; P Scale = Positivity Scale. Results show whether baseline scores (on the left) moderate training effects on various outcomes.

Figure S1

Individual Change in Symptoms of Depression From Baseline to Follow−Up after Six Month

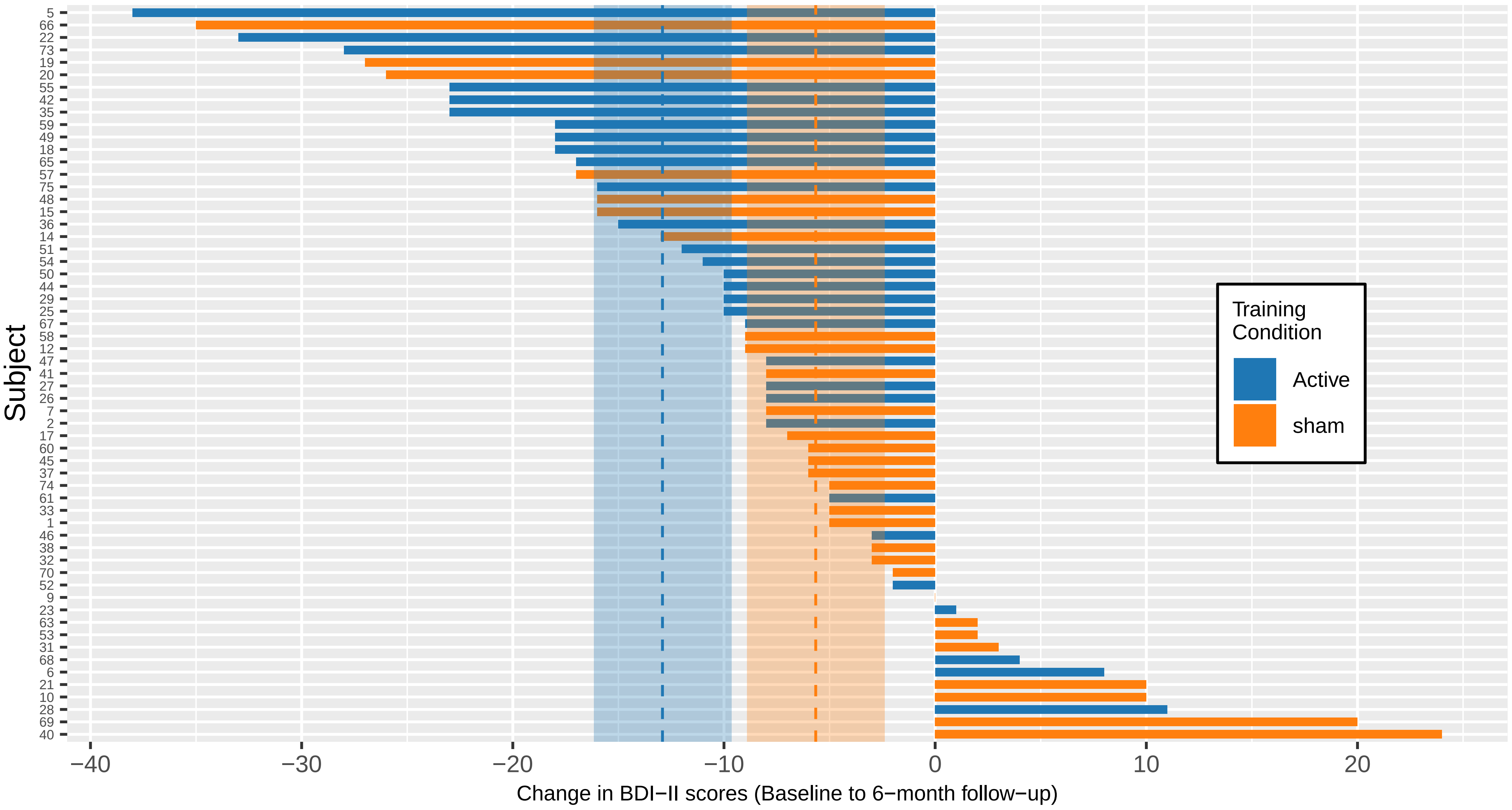

Note. BDI−II = Beck Depression Inventory Second Edition; Bars depict individual changes in depression symptoms for each participant.
Dashed lines indicate model's predicted average reduction in symptoms for each condition, with shaded areas representing the 95% confidence interval.

Figure S2

Model−Based Change Trajectories in Approach−avoidance Bias (Reaction Time)

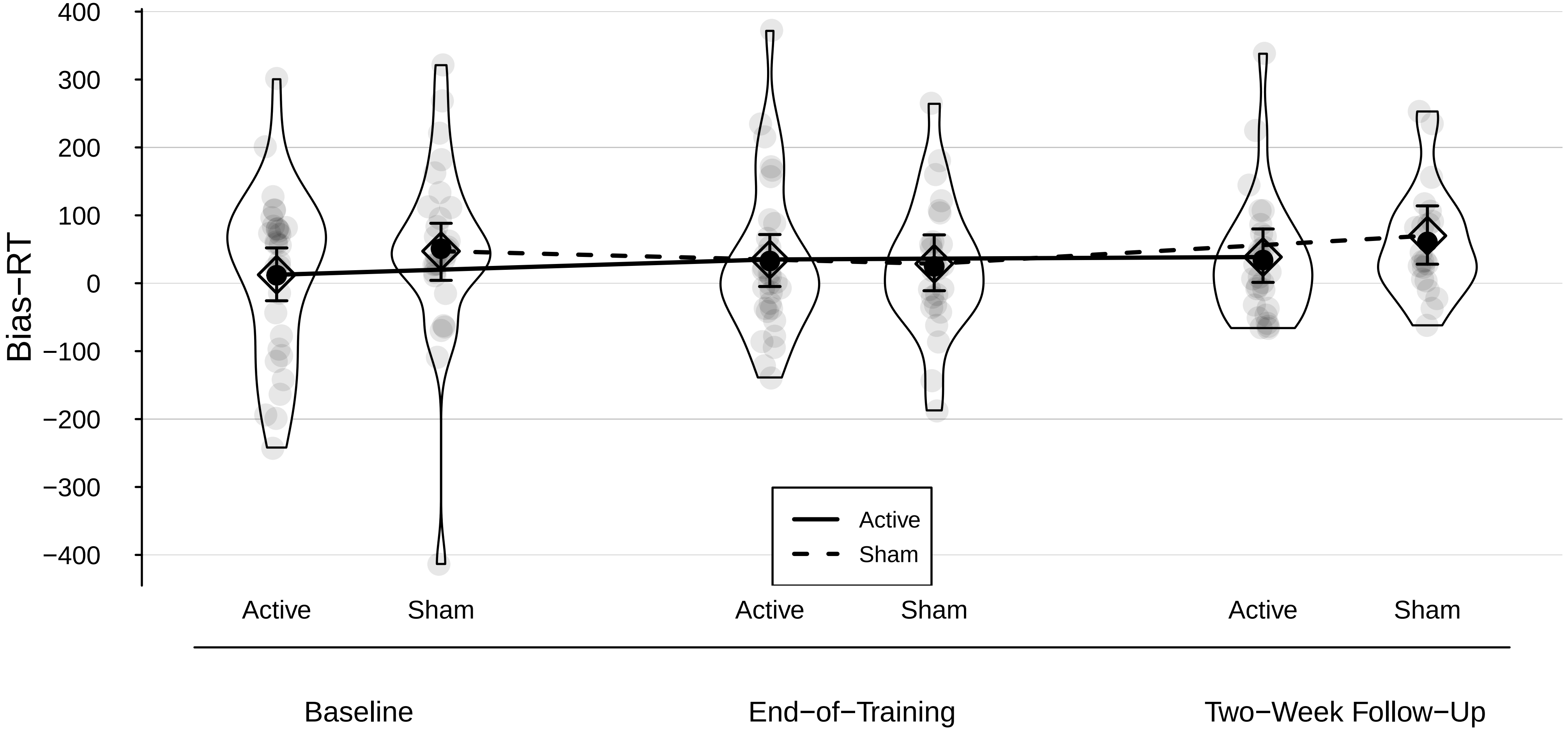

Note. Bias−RT = Approach−avoidance Bias (Reaction Time); Grey dots represent individual data points.
Solid black dots show empirical means. Diamonds show model−based estimates with 95% CIs.
Bean width reflects distribution.

Figure S3

Model−Based Change Trajectories in Approach−avoidance Bias (Response Force)

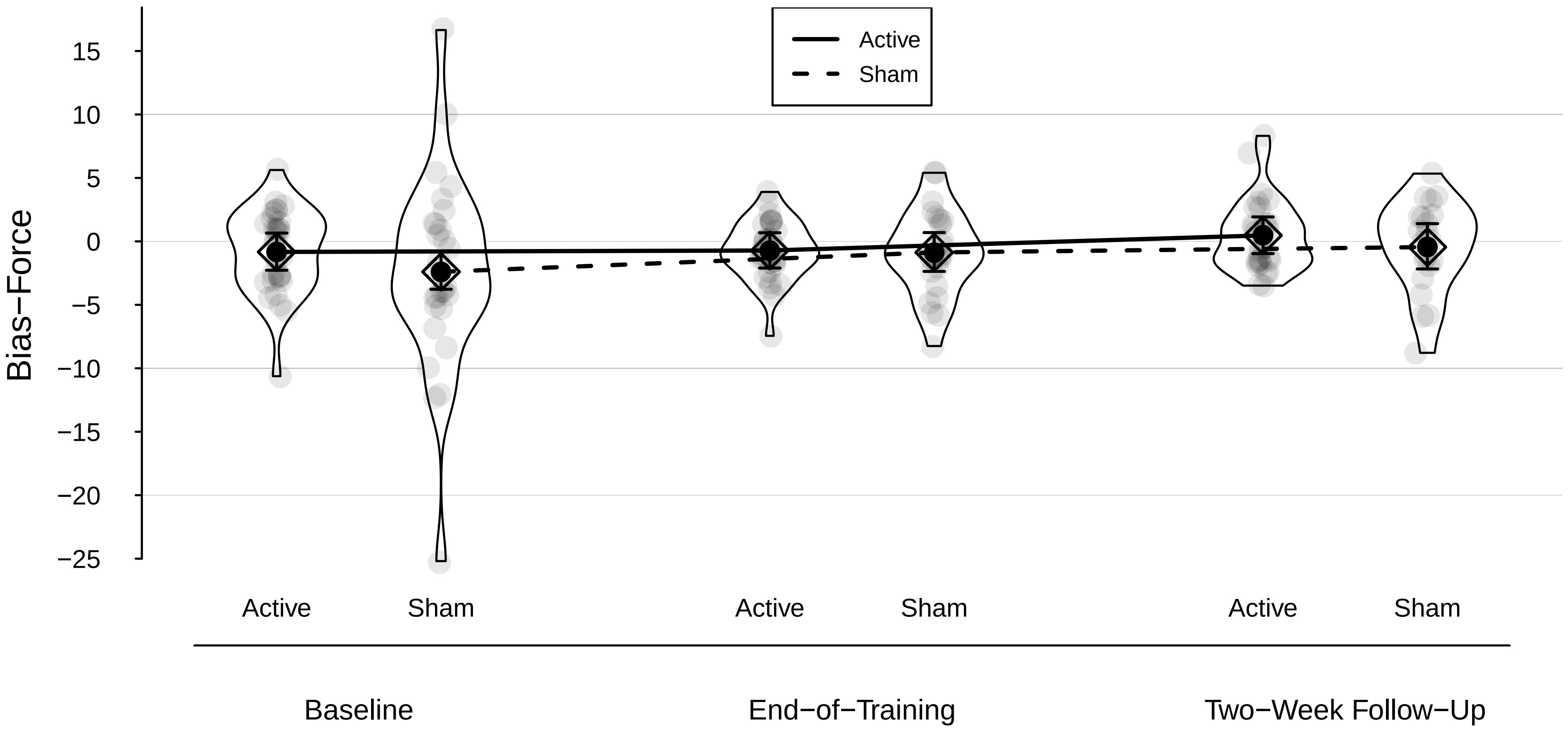

Note. Bias−Force = Approach−avoidance Bias (Response Force); Grey dots represent individual data points.
Solid black dots show empirical means. Diamonds show model−based estimates with 95% CIs.
Bean width reflects distribution.

Figure S4

Model−Based Change Trajectories in Symptoms of Anhedonia

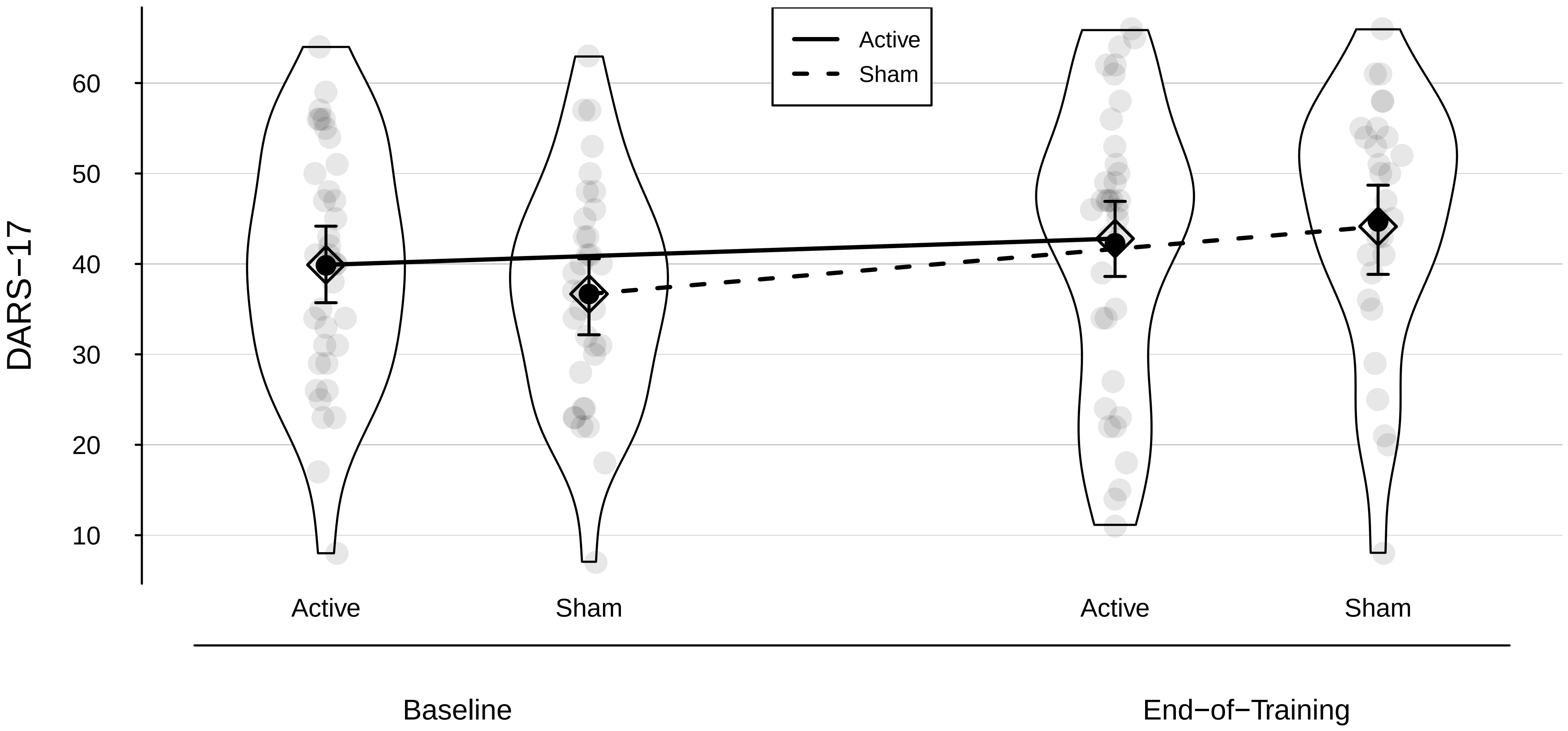

Note. DARS−17 = Dimensional Anhedonia Rating Scale 17; Grey dots represent individual data points.
Solid black dots show empirical means. Diamonds show model−based estimates with 95% CIs.
Bean width reflects distribution.

Figure S5

Model−Based Change Trajectories in Positivity

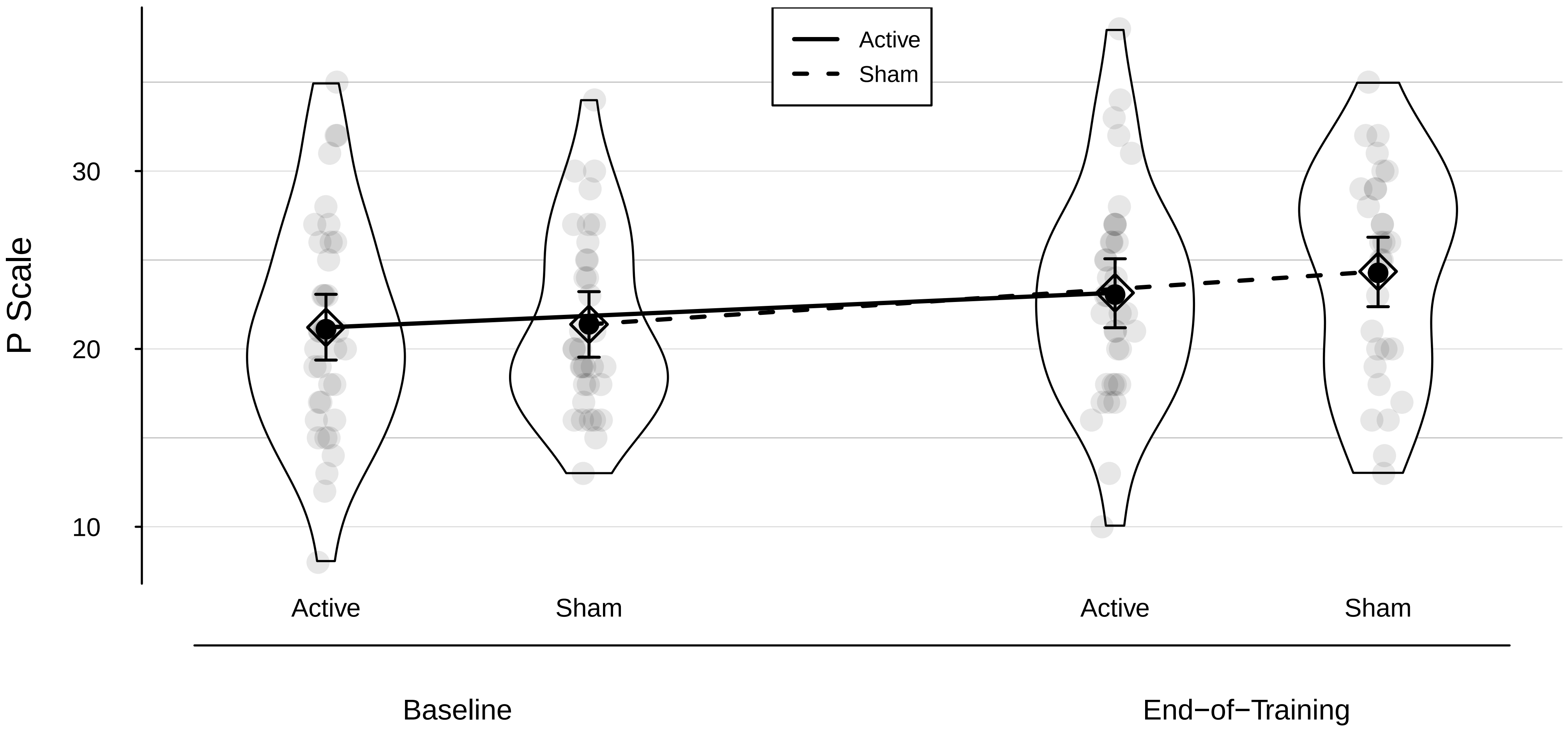

Note. P Scale = Positivity Scale; Grey dots represent individual data points.
Solid black dots show empirical means. Diamonds show model−based estimates with 95% CIs.
Bean width reflects distribution.

Figure S8

Model−Based Change Trajectories in Symptoms of Depression in the Complete Case Analysis

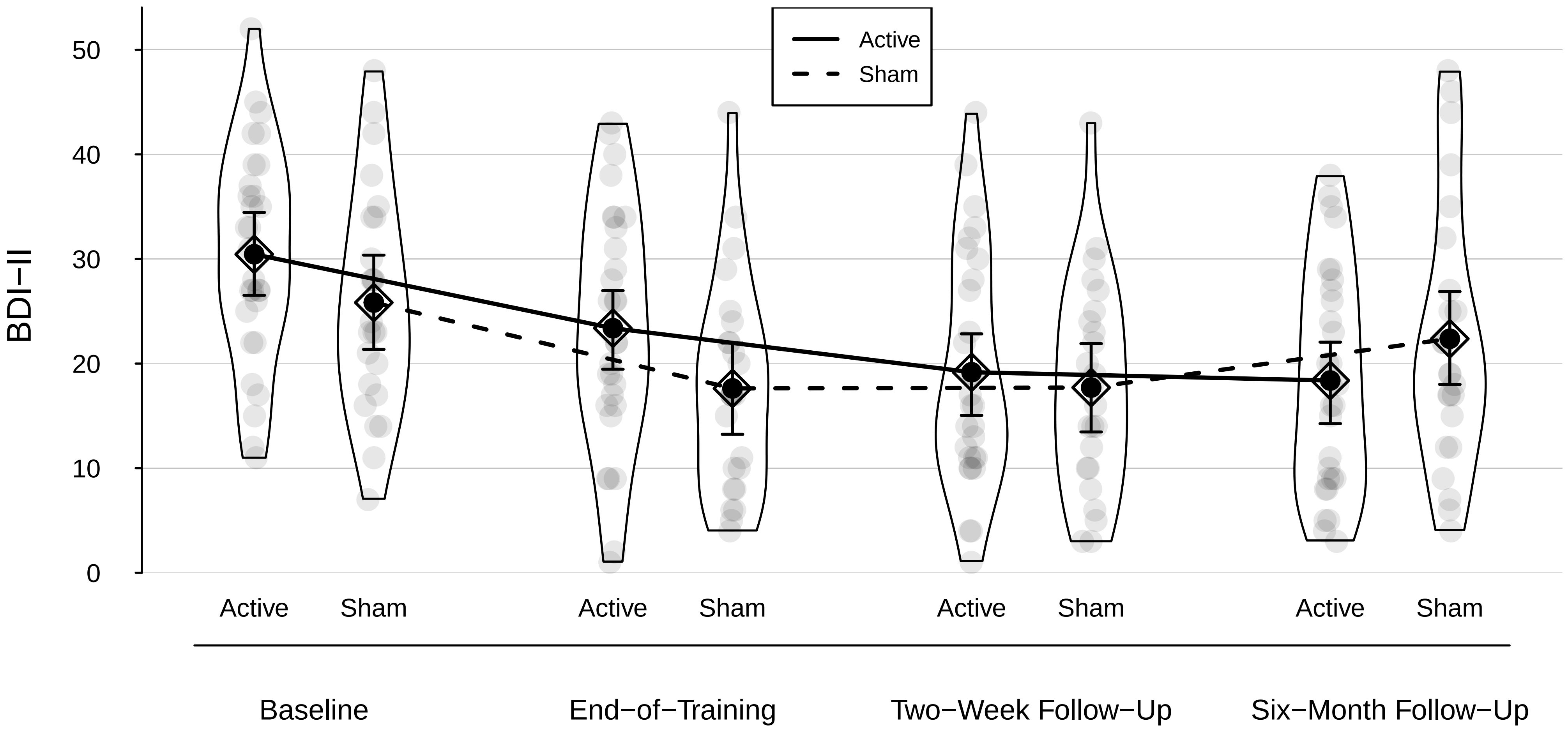

Note. BDI−II = Beck Depression Inventory II; Grey dots represent individual data points.
Solid black dots show empirical means. Diamonds show model−based estimates with 95% CIs.
Bean width reflects distribution.

Figure S9

Model−Based Change Trajectories in Approach−avoidance Bias (Reaction Time) in the Complete Case Analysis

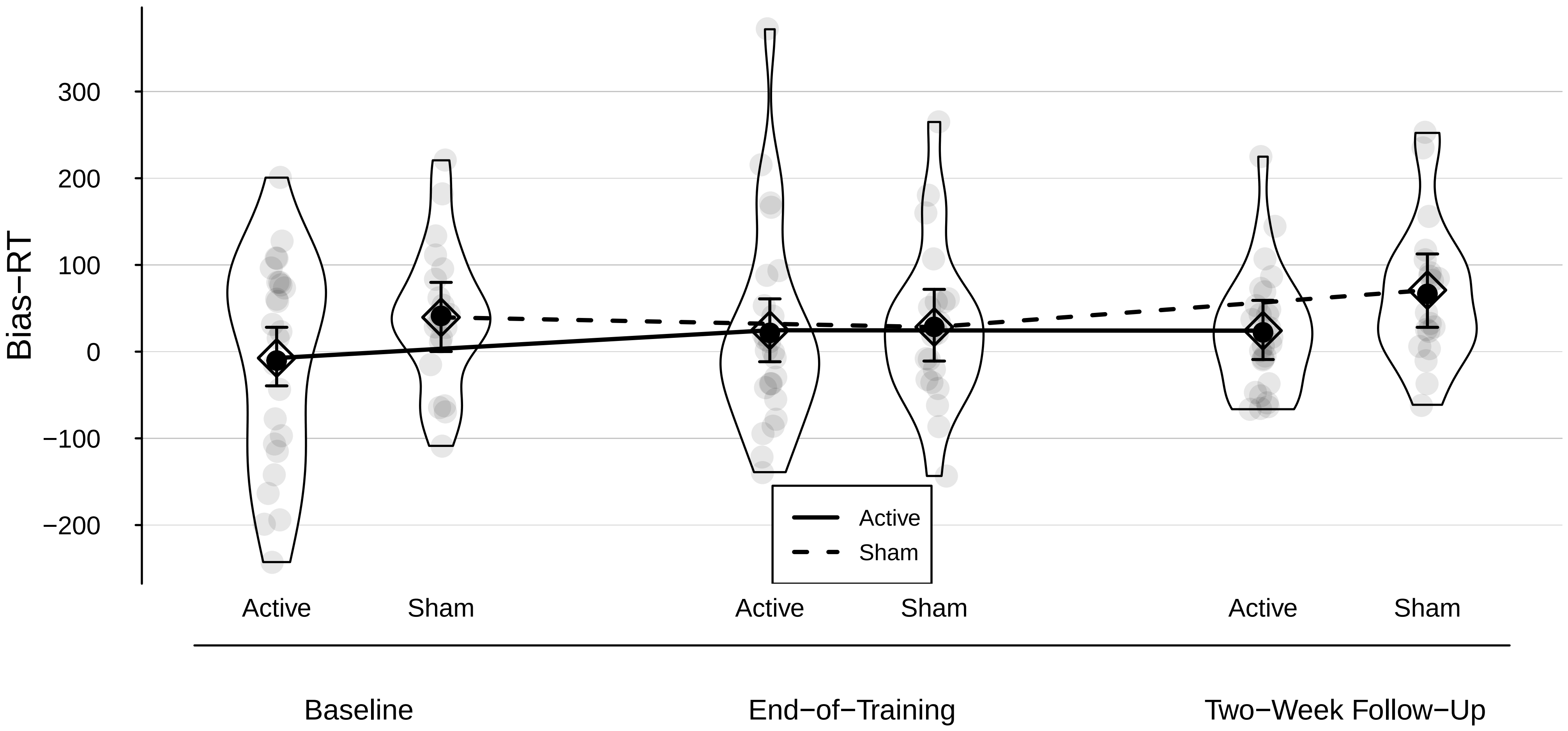

Note. Bias−RT = Approach−avoidance Bias (Reaction Time); Grey dots represent individual data points.
Solid black dots show empirical means. Diamonds show model−based estimates with 95% CIs.
Bean width reflects distribution.

Figure S10

Model−Based Change Trajectories in Approach−avoidance Bias (Response Force) in the Complete Case Analysis

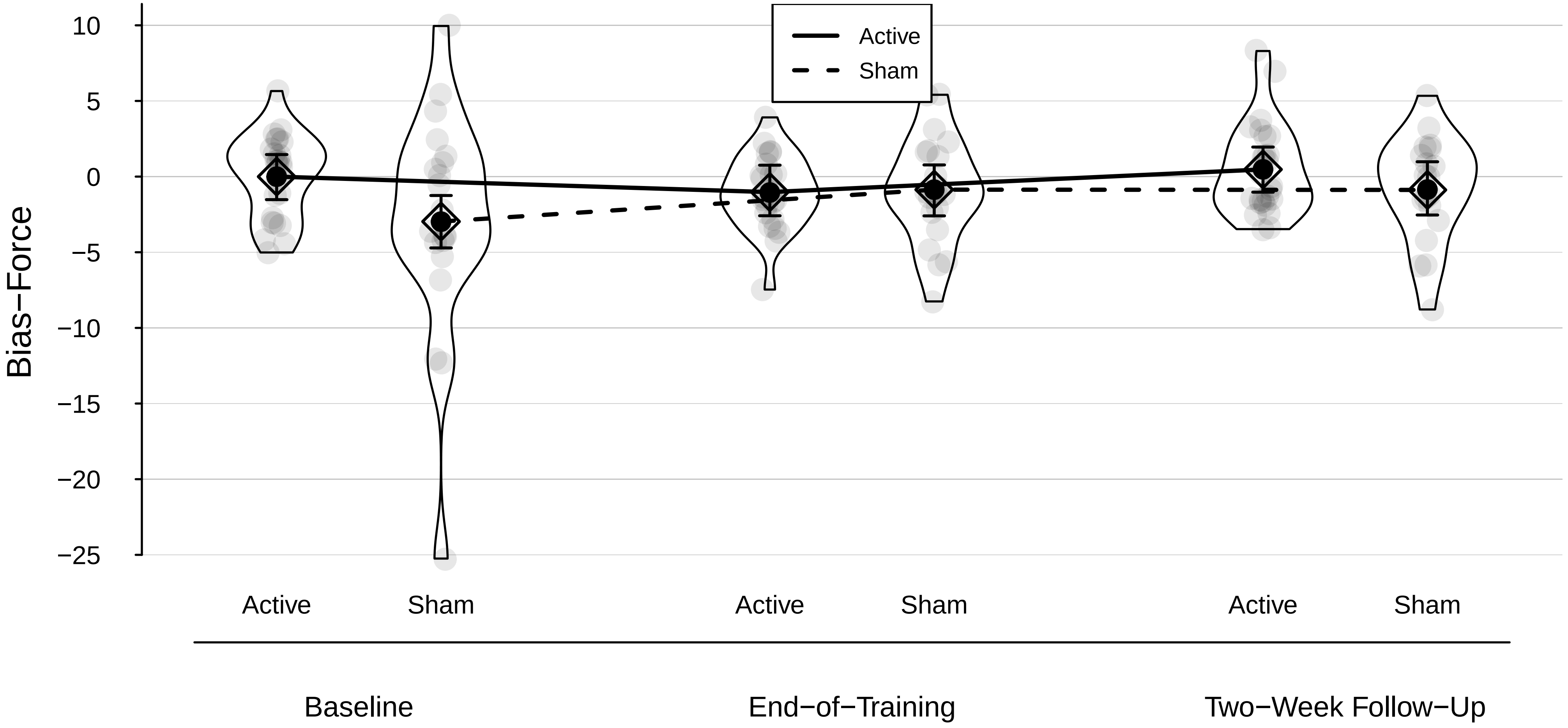

Note. Bias−Force = Approach−avoidance Bias (Response Force); Grey dots represent individual data points.
Solid black dots show empirical means. Diamonds show model−based estimates with 95% CIs.
Bean width reflects distribution.

Figure S11

Model−Based Change Trajectories in Symptoms of Anhedonia in the Complete Case Analysis

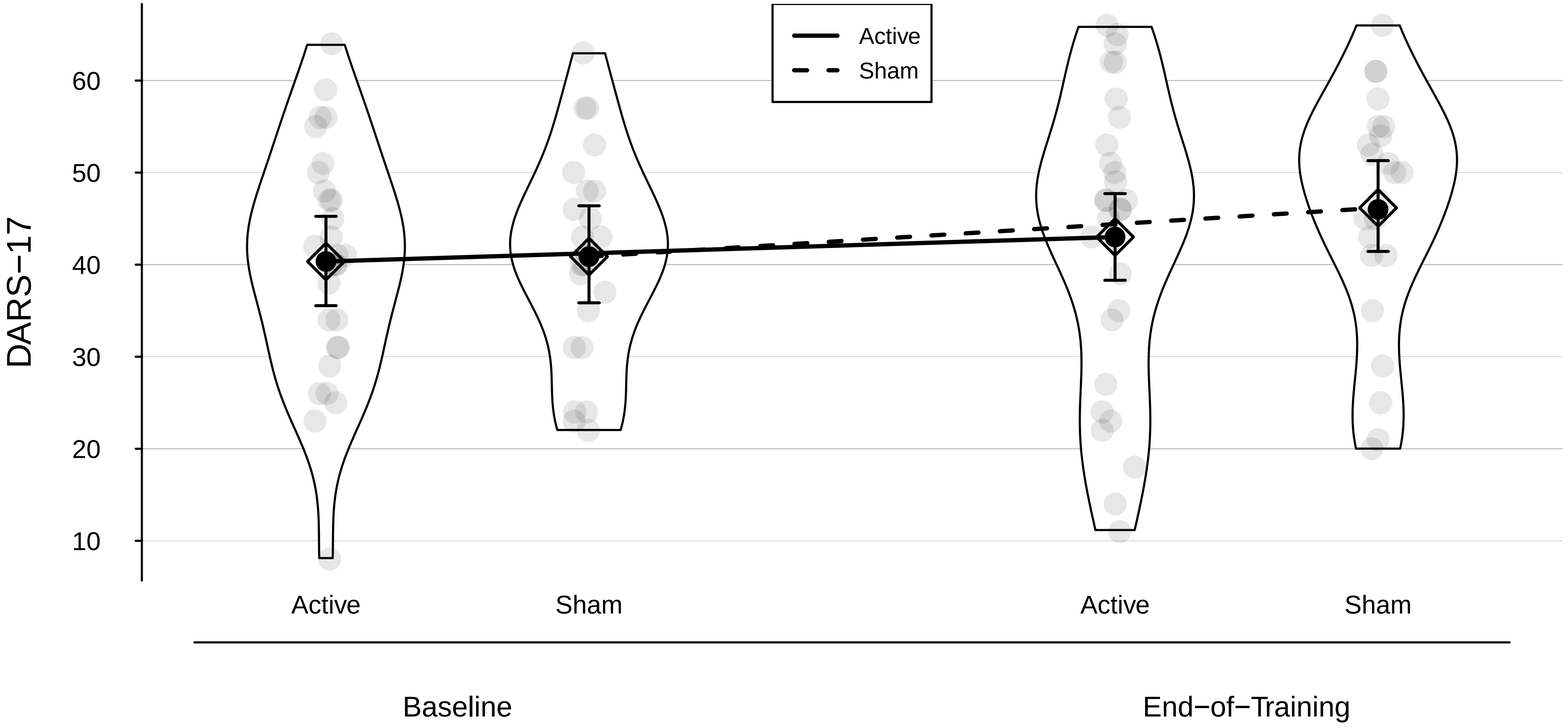

Note. DARS−17 = Dimensional Anhedonia Rating Scale 17; Grey dots represent individual data points.
Solid black dots show empirical means. Diamonds show model−based estimates with 95% CIs.
Bean width reflects distribution.

Supplement References

Baayen, R. H., Davidson, D. J., & Bates, D. M. (2008). Mixed-effects modeling with crossed random effects for subjects and items. *Journal of Memory and Language*, *59*(4), 390–412. https://doi.org/10.1016/j.jml.2007.12.005

Barr, D. J., Levy, R., Scheepers, C., & Tily, H. J. (2013). Random effects structure for confirmatory hypothesis testing: Keep it maximal. *Journal of Memory and Language*, *68*(3), 255–278. https://doi.org/10.1016/j.jml.2012.11.001

Bates, D., Mächler, M., Bolker, B., & Walker, S. (2014). *Fitting Linear Mixed-Effects Models using Lme4* (arXiv:1406.5823). arXiv. https://doi.org/10.48550/arXiv.1406.5823

Davison, A. C., & Hinkley, D. V. (1997). *Bootstrap Methods and Their Application.* Cambridge University Press.

Faul, F., Erdfelder, E., Buchner, A., & Lang, A.-G. (2009). Statistical power analyses using G* Power 3.1: Tests for correlation and regression analyses. *Behavior Research Methods*, *41*(4), 1149–1160.

Feingold, A. (2009). Effect sizes for growth-modeling analysis for controlled clinical trials in the same metric as for classical analysis. *Psychological Methods*, *14*(1), 43–53. https://doi.org/10.1037/a0014699

Hayes, A. F., & Rockwood, N. J. (2017). Regression-based statistical mediation and moderation analysis in clinical research: Observations, recommendations, and implementation. *Behaviour Research and Therapy*, *98*, 39–57. https://doi.org/10.1016/j.brat.2016.11.001

Hedeker, D., & Gibbons, R. D. (1997). Application of random-effects pattern-mixture models for missing data in longitudinal studies. *Psychological Methods*, *2*(1), 64–78. https://doi.org/10.1037/1082-989X.2.1.64

Kahveci, S., Bathke, A. C., & Blechert, J. (2025). Reaction-time task reliability is more accurately computed with permutation-based split-half correlations than with Cronbach's alpha. *Psychonomic Bulletin & Review*, *32*(2), 652–673. https://doi.org/10.3758/s13423-024-02597-y

Kahveci, S., Rinck, M., Van Alebeek, H., & Blechert, J. (2023). How pre-processing decisions affect the reliability and validity of the approach–avoidance task: Evidence from simulations and multiverse analyses

with six datasets. *Behavior Research Methods*, *56*(3), 1551–1582. https://doi.org/10.3758/s13428-023-02109-1

Kuznetsova, A., Brockhoff, P. B., & Christensen, R. H. B. (2017). lmerTest Package: Tests in Linear Mixed Effects Models. *Journal of Statistical Software*, *82*, 1–26. https://doi.org/10.18637/jss.v082.i13

Little, R. J. A. (1988). A Test of Missing Completely at Random for Multivariate Data with Missing Values. *Journal of the American Statistical Association*, *83*(404), 1198–1202. https://doi.org/10.1080/01621459.1988.10478722

Luke, S. G. (2017). Evaluating significance in linear mixed-effects models in R. *Behavior Research Methods*, *49*(4), 1494–1502. https://doi.org/10.3758/s13428-016-0809-y

Team, R. C. (2020). *R: A Language and Environment for Statistical Computing*. R Foundation for Statistical Computing.

Tingley, D., Yamamoto, T., Hirose, K., Keele, L., & Imai, K. (2014). Mediation: R package for causal mediation analysis. *UCLA Statistics/American Statistical Association*.

Zech, H. G., Gable, P., van Dijk, W. W., & van Dillen, L. F. (2022). Test-retest reliability of a smartphone-based approach-avoidance task: Effects of retest period, stimulus type, and demographics. *Behavior Research Methods*. https://doi.org/10.3758/s13428-022-01920-6

Zech, H. G., Rotteveel, M., van Dijk, W. W., & van Dillen, L. F. (2020). A mobile approach-avoidance task. *Behavior Research Methods*, *52*(5), 2085–2097. https://doi.org/10.3758/s13428-020-01379-3
